# Organic Fluorophores for 1064 nm Excited NIR-II Fluorescence Imaging

**DOI:** 10.3389/fchem.2021.769655

**Published:** 2021-11-15

**Authors:** Wenqi Wang, Xiaowen He, Mingzhi Du, Chen Xie, Wen Zhou, Wei Huang, Quli Fan

**Affiliations:** ^1^ Key Laboratory for Organic Electronics and Information Displays and Jiangsu Key Laboratory for Biosensors, Institute of Advanced Materials (IAM), Jiangsu National Synergetic Innovation Center for Advanced Materials (SICAM), Nanjing University of Posts and Telecommunications, Nanjing, China; ^2^ Frontiers Science Center for Flexible Electronics (FSCFE), MIIT Key Laboratory of Flexible Electronics (KLoFE), Northwestern Polytechnical University, Xi’an, China

**Keywords:** NIR-II fluorescence imaging, 1,064 nm excitation, photothermal therapy, phototheranostics, organic fluorophores

## Abstract

Second near-infrared window (NIR-II) fluorescence imaging has shown great potential in the field of bioimaging. However, the excitation wavelengths of most NIR-II fluorescence dyes are in the first near-infrared (NIR-I) region, which leads to limited imaging depth and resolution. To address such issue, NIR-II fluorescence dyes with 1,064 nm excitation have been developed and applied for *in vivo* imaging. Compared with NIR-I wavelength excited dyes, 1,064 nm excited dyes exhibit a higher tissue penetration depth and resolution. The improved performance makes these dyes have much broader imaging applications. In this mini review, we summarize recent advances in 1,064 nm excited NIR-II fluorescence fluorophores for bioimaging. Two kinds of organic fluorophores, small molecule dye and semiconducting polymer (SP), are reviewed. The general properties of these fluorophores are first introduced. Small molecule dyes with different chemical structures for variety of bioimaging applications are then discussed, followed by the introduction of SPs for NIR-II phototheranostics. Finally, the conclusion and future perspective of this field is given.

## Introduction

As a powerful disease diagnostic method, optical imaging has shown great promise both in the laboratory research and clinical application (Ding et al., 2021) (Xu and Pu, 2021). The two major optical imaging modalities are fluorescence and photoacoustic imaging, and near-infrared (NIR) light (650–900 nm) has been widely used as both excitation and signal source because of its relatively high tissue penetration depth compared with visible light (Cheng et al., 2020a) (Guo et al., 2017) (Huang et al., 2019) (Jiang and Pu, 2018) (Xie et al., 2017) (Wang et al., 2021a). NIR fluorescence imaging has unique advantages, such as high biological safety, high spatial and temporal resolution, high sensitivity and low cost (Li and Pu, 2019) (Lovell et al., 2011) (Qi et al., 2019) (Yang et al., 2018) (Zhang et al., 2020a). Until now, variety of NIR fluorescence probes have been designed for imaging cancer, (Huang et al., 2021) (Zhou et al., 2020a) inflammation (Fan et al., 2021) (Liu et al., 2021) (Yang et al., 2020a) (Cheng et al., 2020b), kidney dysfunction (Huang and Pu, 2020) (Huang et al., 2019) (Chen et al., 2021a), liver injury (Cheng et al., 2021) (Kwon et al., 2018), and thrombus (Lee et al., 2019)^,^ (Wang et al., 2021b)^,^ (Jiang et al., 2021). Although NIR fluorescence imaging has shown great potential, its tissue penetration depth still needs to be improved compared with traditional imaging modalities such as X-ray computed tomography (CT) and magnetic resonance imaging (MRI) (Li et al., 2021) (Miao and Pu, 2018) (Li and Pu, 2019) (Wang et al., 2019a) (Guo et al., 2019). Thus, fluorescence imaging in the second NIR window (NIR-II, 1,000–1,700 nm) has gained more and more attention, as it has both higher tissue penetration depth and spatial resolution than fluorescence imaging in the first NIR (NIR-I) window (Sheng et al., 2018) (Zhou et al., 2020b) (Guo et al., 2019) (Gong et al., 2013).

Until now, different kinds of NIR-II fluorescence probes have been developed for disease diagnosis, including carbon nanotubes (Zhao et al., 2018) (Antaris et al., 2016), small molecule dyes (Zhang et al., 2021) (Zhou et al., 2020c) (Chen et al., 2021b), semiconducting polymers (SPs), and quantum dots(Feng et al., 2021) (Kong et al., 2016) (Li et al., 2015) (Zhang et al., 2019) (Chen et al., 2021c). Among these materials, organic fluorophores including small molecule dyes and SPs have shown great potential owing to their good biocompatibility and high absorption coefficient(Hu et al., 2021) (Tian et al., 2020) (Wang et al., 2020) (Xie et al., 2020) (Yang et al., 2020b) (Zhou et al., 2021) (Ou et al., 2021). However, the excitation wavelengths for most of organic fluorophores are still in the range of NIR-I window. Compared with the most commonly used excitation wavelength 808 nm, 1,064 nm light has both deeper tissue penetration and higher maximum permissible exposure (MPS) intensity (1 vs. 0.33 W/cm^2^)(Zhen et al., 2021) (Zhang et al., 2020b). Such feature makes NIR-II fluorescence imaging under 1,064 nm excitation have a much better imaging depth and resolution than that under 808-nm excitation (Du et al., 2021). However, it is difficult to design and synthesize organic NIR-II fluorescence probes with NIR-II excitation wavelength. Thus, developing such kind of organic fluorophores to further promote the effect of NIR-II fluorescence imaging is of great significance.

The design, preparation, and applications of NIR-II fluorescence probes have been very well summarized by several reviews (Su et al., 2021)^,^ (Zhu et al., 2019)^,^ (Wang et al., 2019b). Therefore, in this mini review, we focus on recent progress in the development of 1,064 nm excited organic NIR-II fluorescence probes. In the first part, the general classification and synthesis of these probes are introduced. Then, different kinds of 1,064-nm excited NIR-II fluorescent small molecule dyes and semiconducting polymers are introduced separately. Finally, a brief summary and the current status as well as potential challenge of this field are discussed.

## General Classification and Properties of Organic Fluorophores

Organic fluorophores can be classified into two types, small molecule dyes and SPs. For small molecule dyes, large π-conjugated backbones or electron donor–acceptor (D-A) structures are required to extend the absorption of these dyes into the NIR-II region. Compared with SPs, small molecule dyes have definite structure and smaller molecular weight. By virtue of such feature, small molecule dyes may be metabolized *via* renal clearance and, thus, have greater potential in clinical translation (Zhang et al., 2021) (Chen et al., 2021b). However, sophisticated synthetic and purification procedures are usually required for small molecule dye preparation owing to their relatively complex structures. In contrast, the synthetic and purification procedures for SPs are much easier. The commonly used reactions for SP preparation are palladium-catalyzed Suzuki and Stille coupling; both of them are well studied and widely applied (Li et al., 2018). By choosing proper electron donor and acceptor monomers, SPs with NIR-II absorption can be readily synthesized. In addition, SPs have better photostability than small molecule dyes (Zhou et al., 2020a). Overall, both small molecule dyes and SPs have their own advantages and have been developed for 1,064 nm NIR-II excitation fluorescence contrast agents.

## Small Molecule Dyes for 1,064 nm NIR-II Excitation Fluorescence Imaging

**FIGURE 1:**
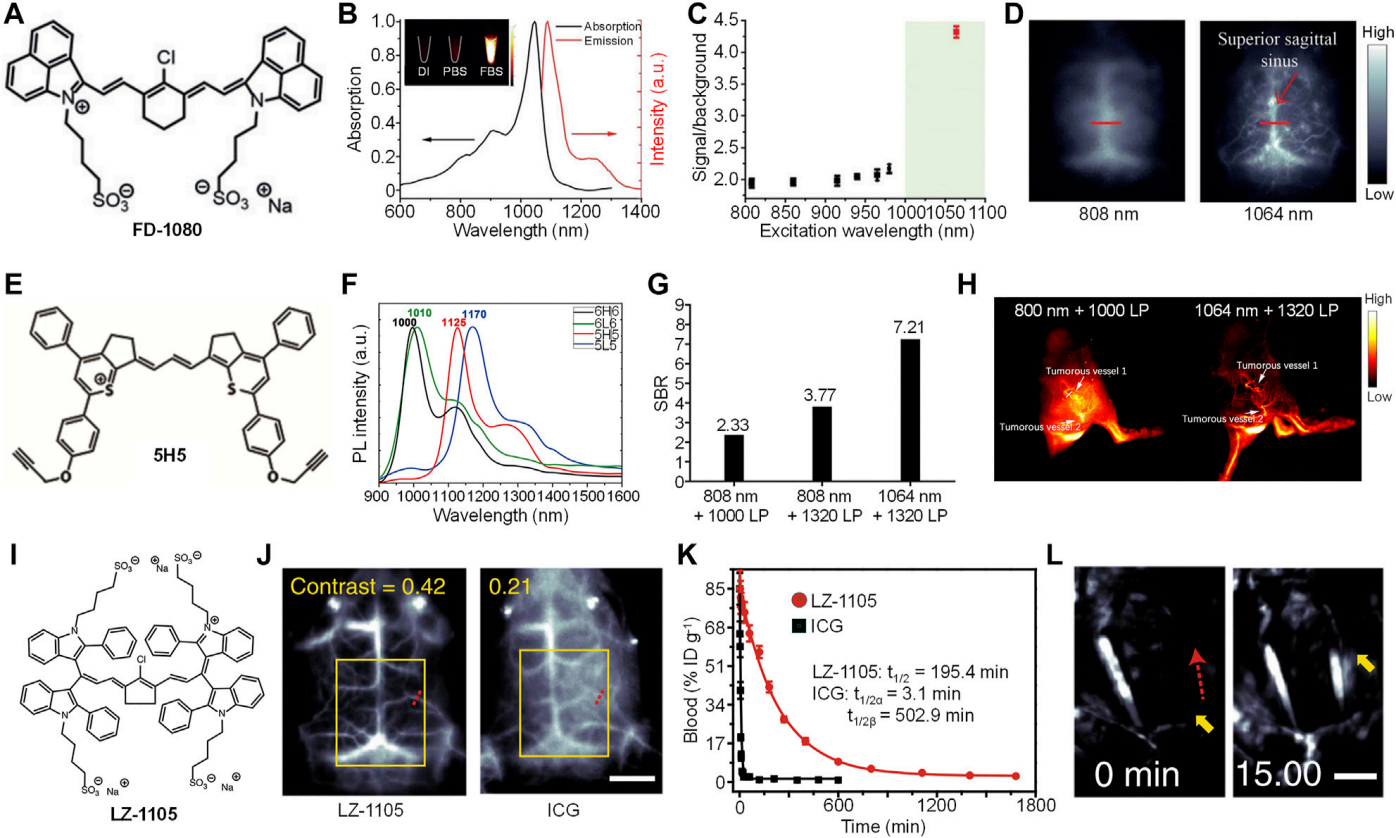
**(A)** Chemical structure of cyanine-based dye (FD-1080). **(B)** Absorption and emission spectra of FD-1080. Inset: Representative second near-infrared window (NIR-II) fluorescence images of FD-1080 in deionized (DI) water, PBS, and fetal bovine serum (FBS) under 1,064 nm excitation. **(C)** The signal-to-background ratio of mouse left hindlimb vasculature injected with FD-1080-FBS under different excitation wavelengths. **(D)** Representative NIR-II fluorescence images of mouse brain vasculature injected with FD-1080-FBS under 808 or 1,064-nm excitation. **(E)** Chemical structure of 5H5. **(F)** Fluorescence spectra of 6H6, 6L6, 5H5, and 5L5. **(G)** The signal-to-background ratio of mouse hindlimb vasculature injected with 5H5 under different excitation wavelengths and filters. **(H)** Representative NIR-II fluorescence images of mouse blood vessels around and within tumor under different excitation wavelengths and filters. **(I)** Chemical structure of LZ-1105 **(J)** Representative NIR-II fluorescence images of mouse brain injected with LZ-1105 or indocyanine green (ICG) under 1,064 and 808 nm excitation, respectively. The scale bar represents 3 mm **(K)** Blood concentration of LZ-1105 and ICG in living mice as a function of time **(L)** Representative NIR-II fluorescence images of LZ-1105-injected mouse carotid artery before and after injection of recombinant tissue plasminogen activator for 15 min. The scale bar represents 3 mm. Adapted from ref. 19–21. Copyright© 2018 Wiley-VCH Verlag GmbH and Co. KGaA, Weinheim, 2019 American Chemical Society and 2020 Springer Nature.

The first example of small molecule dye for 1,064 nm excitation NIR-II fluorescence imaging is reported by Zhang et al. (Ding et al., 2019). They designed and synthesized a cyanine-based dye, FD-1080, with good water solubility (Figure 1A). Compared with the commercially available NIR-I dye indocyanine green (ICG), FD-1080 had a better photostability. FD-1080 showed an NIR-II absorption peak at 1,046 nm, and an emission peak at 1,080 nm under 1,064 nm excitation (Figure 1B). The quantum yield of FD-1080 was determined as 0.31% in ethanol, and can be significantly increased into 5.94% by being encapsulated with fetal bovine serum (FBS). Tissue penetration study indicated that, under 1,064 nm excitation, the NIR-II fluorescence signal of FD-1080 can resolve the edge of capillary tube even under 5-mm phantom, which was much deeper than excitation under NIR-I light. The *in vivo* imaging performance of FD-1080 was first evaluated by NIR-II fluorescence imaging of left hindlimb of mice. FD-1080 was encapsulated by FBS to give FD-1080-FBS complex, and was intravenously (i.v.) injected into the mice. The results showed that under 1,064 nm excitation, the signal-to-background ratio (SBR) of hindlimb vessel (4.32) was much higher than that under other shorter wavelengths (ranging from 1.9 to 2.2) (Figure 1C). The resolved blood vessel under 1,064 nm excitation had a full width at half-maximum (FWHM) of 0.47 mm. Similarly, under 1,064 nm excitation, NIR-II fluorescence signal of FD-1080-FBS complex can clearly resolve the sagittal sinus vessel of mouse with a FWHM of 0.65 mm, while such resolution can only reach 1.43 mm under 808 nm excitation (Figure 1D). Furthermore, the FD-1080-FBS complex can quantify the respiratory rate of mice by real-time NIR-II fluorescence imaging of respiratory craniocaudal motion of the liver and, thus, differentiate awake and anesthetized mice.

To prepare high-performance NIR-II fluorescence dyes, Hong et al. synthesized four NIR-II fluorophores with alkyne groups, which can be conjugated with targeting groups for cancer imaging. (Li et al., 2020). Among them, 5H5 had the highest quantum yield of 2.6% (Figure 1E). The maximum absorption of 5H5 was longer than 1,000 nm, with a maximum emission of 1,125 nm (Figure 1F). As 5H5 had the highest quantum yield, it was then applied for bioimaging applications. Owing to the two alkyne groups in the structure of 5H5, azide-functionalized cyclic arginylglycylaspartic acid (cRGD) can be linked with 5H5 to give 5H5-PEG_8_-cRGD. Because of the conjugation of PEG, the water solubility of 5H5 was improved, thus, increasing the blood circulation of 5H5-PEG_8_-cRGD. After i.v. injection of 5H5-PEG_8_-cRGD, the main blood vessel of mice can be clearly observed by NIR-II fluorescence imaging. Under 808 nm excitation, the imaging under 1,320 long-pass (LP) filter had a better SBR than that under 1,000 LP filter (3.8 vs. 2.3). An even higher SBR (7.2) was detected for the imaging under 1,064 nm excitation with 1,320 LP filter, which indicated that longer excitation and emission wavelength had lower background signal (Figure 1G). In addition, such imaging condition showed a much better resolution than that under 808-nm excitation. The 5H5-PEG_8_-cRGD was then applied for tumorous microvasculature imaging. After i.v. injection of 5H5-PEG_8_-cRGD, tumorous vessels 1 and 2 were clearly delineated by NIR-II fluorescence imaging both under 808 and 1,064-nm excitation (Figure 1H). Compared with 808-nm excitation, NIR-II fluorescence imaging under 1,064 nm showed a higher resolution. Blood vessels 1 and 2 were measured as 318 and 516 μm under 1,064 nm excitation, respectively, whereas those measured as 407 and 587 μm were under 808-nm excitation. The 5H5 nanoparticles showed good tumor-targeting capability. At t = 48 h post-injection, tumor tissue had an SBR of 3.2 under 808-nm excitation, while such SBR can increase to 6.5 under 1,064 nm excitation. In addition, 5H5-PEG_8_-cRGD can effectively accumulate into U87MG glioma tumors with integrin α_v_β_3_ overexpression. The NIR-II fluorescence imaging data also confirmed that the tumor site had a higher SBR under 1,064 nm excitation than that under 808 nm excitation.

To achieve *in vivo* dynamic vascular imaging in the NIR-II window, Zhang et al. designed a long blood half-life small molecule dye, LZ-1105 (Figure 1I) (Jiang et al., 2017). LZ-1105 had absorption (1,041 nm) and emission (1,105 nm) wavelength both in the NIR-II window, and the quantum yield of LZ-1105 was determined as 1.69%. In addition, LZ-1105 had a better photostability than ICG. The tissue penetration depth of NIR-II fluorescence signal of LZ-1105 can reach 6 mm, which was much deeper than that of ICG excited under 808 nm. The *in vivo* NIR-II fluorescence imaging capability of LZ-1105 was then evaluated. Cerebral vessel imaging showed that NIR-II fluorescence signal of LZ-1105 under 1,064-nm excitation can clearly delineate the blood vessel with very high resolution, which was 8.4-fold higher than that of ICG under 808-nm excitation. In addition, the contrast of LZ-1105-injected mice (0.42) was twofold higher than that of ICG-injected mice (0.21) (Figure 1J), demonstrating the higher resolution and contrast for LZ-1105. LZ-1105 showed a long blood circulation time, and the vasculature of mice can be clearly visualized even after 12 h post-injection. The SBR of blood vessel was higher than five at t = 4 h post-injection. For ICG, the blood vessels can only be distinguished at 5 min post-injection. The blood circulation time study indicated that LZ-1105 had a long blood half-life of 195.4 min, while such time was only 3.1 min for ICG (Figure 1K). Owing to the ultralong blood circulation time, LZ-1105 can be applied for real-time imaging of ischemic reperfusion in hindlimb. After i.v. injection of LZ-1105, the recovery of blood flow can be indicated by the NIR-II fluorescence signal of LZ-1105. The process of thrombolysis can also be monitored by LZ-1105. After i.v. injection of LZ-1105, the blood vessel with thrombus showed no NIR-II fluorescence signal because of the occlusion of blood flow. After injection of rt-PA to destruct the blood clot, the fluorescence signal of such blood vessel recovered gradually, and such signal reached its maximum at t = 15 min post-injection of rt-PA, indicating the success of thrombolysis (Figure 1l). LZ-1105 can also be used for monitoring the opening and recovery of blood brain barrier (BBB). When BBB was opened, an obvious bright spot can be observed in the brain tissue, which can be attributed to the leakage of LZ-1105 into brain tissue due to the opening of BBB. After the recovery of BBB, such bright spot gradually disappeared. Such phenomenon demonstrated that LZ-1105 can real-time monitor the status of BBB.

Overall, all of these small molecule dyes showed good NIR-II fluorescence imaging effect under 1,064 nm excitation. They had both higher tissue penetration depth and imaging resolution than their counterparts under 808 nm excitation. In addition, their quantum yields were relatively high, all of them were higher than 1%, and the highest can reach nearly 6% with the help of FBS. Owing to such feature, they can be applied for imaging blood vessel, tumor, respiratory craniocaudal motion, BBB and so on.

## Semiconducting Polymers for 1,064-nm Second Near-Infrared Window Excitation Fluorescence Imaging

Compared with small molecule dyes, SPs with absorption longer than 1,000 nm are readily synthesized, as SPs usually have larger molecular weight, which leads to larger π-conjugated backbone and longer absorption wavelength. (Jiang et al., 2020). However, most of them showed almost no fluorescence signal but good photothermal conversion efficiency.(Xu et al., 2021) (Huang et al., 2020) Thus, developing SPs with high NIR-II fluorescence quantum yield under 1,064 nm excitation is still a great challenge.

**FIGURE 2:**
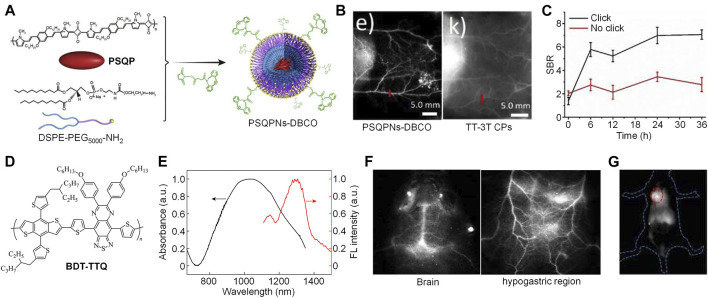
**(A)** Schematic illustration of preparation of PSQPNs-DBCO. **(B)** NIR-II fluorescence imaging of blood vessels by PSQPNs-DBCO and TT-3T CPs. **(C)** Signal-to-background ratio (SBR) of tumor tissue of mice with different treatments. **(D)** Chemical structure of BDT-TTQ. **(E)** Absorption and emission spectra of BDT-TTQ NPs in water. **(F)** NIR-II fluorescence imaging of brain and hypogastric region of BDT-TTQ NPs-injected mice. **(G)** NIR-II fluorescence imaging of tumor after i.v. injection of BDT-TTQ NPs for 12 h. The red circle indicates the location of tumor. Adapted from ref. 16 and 24. Copyright© 2020 Elsevier and 2020 Chinese Chemical Society.

Our group successfully synthesized a squaraine-based SP (PSQP) by using squaraine as the electron acceptor unit and 1,4-bis [2-(1-methylpyrrol-2-yl)vinyl]-2,5-didodecyloxybenzene (BP) as the electron donor unit. PSQP was encapsulated into DSPE-PEG_5000_-NH_2_ to form water-soluble PSQP nanoparticles (PSQPNs), and an alkynyl group-containing compound DBCO-NHS was conjugated onto the surface of PSQPNs to give PSQPNs-DBCO (Figure 2A). PSQPNs-DBCO had a spherical morphology with a hydrodynamic size of 172.4 nm. PSQPNs-DBCO had a maximum absorption of about 900 nm, and an NIR-II emission at around 1,300 nm. In addition, PSQPNs-DBCO had good photostability, wherein its fluorescence signal remained almost the same even under continuous 1,064-nm light irradiation for 1 h. The *in vivo* NIR-II fluorescence imaging effect of PSQPNs-DBCO was compared with an 808-nm excited SPN, TT-3T CPs. PSQPNs-DBCO-injected mice showed a much higher imaging resolution than TT-3T CPs-injected mice for blood vessels (Figure 2B). The photothermal effect of PSQPNs-DBCO was then tested. The highest photothermal temperature of PSQPNs-DBCO under 1,064-nm light irradiation can reach 86.5°C, while such temperature can only reach 48 C under 808-nm light irradiation. The good photothermal effect of PSQPNs-DBCO made it suitable for NIR-II fluorescence imaging-guided photothermal therapy (PTT). As the surface of PSQPNs-DBCO had alkynyl groups, they can effectively conjugate with 1,3,4,6-tetra-O-acetyl-N-azidoacetyl-D-mannosamine (Ac4ManNAz)-incubated cells via biorthogonal click chemistry. PSQPNs-DBCO showed a much higher cellular uptake for Ac4ManNAz-incubated cancer cells than PSQPNs, which indicated that the click strategy can effectively increase the cellular uptake. The effect of click strategy for *in vivo* tumor targeting was then evaluated. Tumor-bearing mice were pre-injected with Ac4ManNAz for 3 days. After that, PSQPNs-DBCO or PSQPNs were i.v. injected into mice, and the NIR-II fluorescence images of mice were captured at designated time points. For PSQPNs-DBCO-injected mice, an obvious higher SBR was observed in the tumor site than PSQPNs-injected mice (Figure 2C). Such phenomenon can be attributed to the click reaction between PSQPNs-DBCO and azide groups expressed on the surface of tumor cells, which significantly increased the tumor accumulation.

Recently, our group reported another example of NIR-II fluorescent SP, BDT-TTQ, with 1,064 nm excitation for tumor imaging (Figure 2D) (Cheng and Pu, 2020). BDT-TTQ was prepared *via* Stille coupling, and its chemical structure and molecular weight were studied by ^1^H NMR and GPC, respectively. To endow BDT-TTQ with good water solubility, an amphiphilic copolymer F127 was used to encapsulate BDT-TTQ to give BDT-TTQ nanoparticles (BDT-TTQ NPs). BDT-TTQ NPs had a hydrodynamic size of 62 nm with low polydispersity. BDT-TTQ NPs had strong absorption at 1,064 nm, and its emission wavelength was longer than 1,300 nm (Figure 2E). BDT-TTQ NPs had good photothermal efficiency. For the concentration of 0.1 mg/ml, the temperature of BDT-TTQ NPs solution can reach higher than 60 C under 1,064-nm laser irradiation (1 W/cm^2^). The NIR-II fluorescence imaging depth of BDT-TTQ NPs was determined as 6 mm by covering chicken breast tissue onto BDT-TTQ NPs solutions. Owing to the high resolution of NIR-II fluorescence imaging, BDT-TTQ NPs was applied for *in vivo* blood vessel imaging. Under 1,064 nm light excitation, the blood vessels in the brain and hypogastric region can be clearly distinguished, and the SBR of blood vessels can reach 5 (Figure 2F). *In vitro* cytotoxicity study indicated that BDT-TTQ NPs had good biocompatibility without laser irradiation. In contrast, obvious cytotoxicity was observed for BDT-TTQ NPs-treated HeLa cells under 1,064 nm laser irradiation for 5 min, which showed that BDT-TTQ NPs had good photothermal efficacy against tumor cells. BDT-TTQ NPs was then applied for *in vivo* NIR-II fluorescence imaging-guided PTT. After i.v. injection of BDT-TTQ NPs into tumor-bearing mice, the NIR-II fluorescence signal at tumor site gradually increased, indicating that BDT-TTQ NPs can effectively accumulate into tumor. Such signal reached the maximum at t = 12 h post-injection (Figure 2G). At such time point, the NIR-II fluorescence signal of BDT-TTQ NPs can clearly delineate the contour of tumor. *In vivo* PTT results showed that the tumor growth can be significantly inhibited for BDT-TTQ NPs-treated mice under 1,064-nm laser irradiation. Overall, BDT-TTQ NPs was a good 1,064-nm excited NIR-II phototheranostic system.

Because SPs had large molecular weight and poor water solubility, encapsulating SPs with amphiphilic copolymer to give water dispersible SPNs was a rational choice for biological applications. Thus, these probes were mostly in the nanoscale, which were very suitable for tumor imaging owing to the enhanced permeation and retention (EPR) effect. However, SPNs usually had lower NIR-II fluorescence quantum yield than small molecule dyes, and such disadvantage limited the application of SPNs. Thus, improving the quantum yield of SPNs is highly demanded.

## Discussion

We herein summarized organic fluorophores including small molecular dyes and SPs for 1,064-nm NIR-II excitation fluorescence imaging. Compared with NIR-II fluorescence dyes under 808-nm excitation, these dyes have a deeper tissue penetration and higher imaging resolution because of the longer excitation wavelength. The developed 1,064-nm excited small molecular dyes have good water solubility and photostability, and can be applied for NIR-II fluorescence imaging of blood vessel, thrombus, tumor, and respiratory craniocaudal motion. In contrast, SPs have poor water solubility, and amphiphilic copolymers are utilized to encapsulate these SPs to give water soluble SPNs. These SPNs not only show satisfactory NIR-II fluorescence signal under 1,064-nm laser, but also have good photothermal effect. Thus, SPNs can be applied for *in vivo* 1,064-nm excited NIR-II fluorescence imaging-guided PTT.

Although 1,064 nm excited organic fluorophores have been developed for NIR-II fluorescence imaging with high imaging depth and resolution, some critical issues are still needed to be resolved to further promote their biological applications. Compared with 808-nm excited dyes, the quantum yield of 1,064 nm excited dyes are relatively low. Thus, developing organic dyes with novel chemical structures is required for screening NIR-II fluorescence fluorophores with high quantum yield under 1,064-nm excitation. On the other hand, activatable probes usually have higher SBR and specificity compared with “always-on” probes because their signals are only activated in the presence of specific biomarkers or microenvironments (Li et al., 2019) (Meng et al., 2018). Until now, the excitation wavelengths of reported activatable NIR-II fluorescence probes are still limited in the NIR-I window. Therefore, to further improve the sensitivity and selectivity of 1,064-nm excited NIR-II fluorescence dyes, endowing these dyes with specific environmental responsiveness is of great significance.
